# Estimating Population Size for Capercaillie (*Tetrao urogallus* L.) with Spatial Capture-Recapture Models Based on Genotypes from One Field Sample

**DOI:** 10.1371/journal.pone.0129020

**Published:** 2015-06-18

**Authors:** Pierre Mollet, Marc Kéry, Beth Gardner, Gilberto Pasinelli, J. Andrew Royle

**Affiliations:** 1 Swiss Ornithological Institute, Seerose 1, CH-6204, Sempach, Switzerland; 2 North Carolina State University, Raleigh, North Carolina, 27695, United States of America; 3 U. S. Geological Survey, Patuxent Wildlife Research Center, Laurel, Maryland, 20708, United States of America; University of KwaZulu-Natal, SOUTH AFRICA

## Abstract

We conducted a survey of an endangered and cryptic forest grouse, the capercaillie *Tetrao urogallus*, based on droppings collected on two sampling occasions in eight forest fragments in central Switzerland in early spring 2009. We used genetic analyses to sex and individually identify birds. We estimated sex-dependent detection probabilities and population size using a modern spatial capture-recapture (SCR) model for the data from pooled surveys. A total of 127 capercaillie genotypes were identified (77 males, 46 females, and 4 of unknown sex). The SCR model yielded atotal population size estimate (posterior mean) of 137.3 capercaillies (posterior sd 4.2, 95% CRI 130–147). The observed sex ratio was skewed towards males (0.63). The posterior mean of the sex ratio under the SCR model was 0.58 (posterior sd 0.02, 95% CRI 0.54–0.61), suggesting a male-biased sex ratio in our study area. A subsampling simulation study indicated that a reduced sampling effort representing 75% of the actual detections would still yield practically acceptable estimates of total size and sex ratio in our population. Hence, field work and financial effort could be reduced without compromising accuracy when the SCR model is used to estimate key population parameters of cryptic species.

## Introduction

Capture-mark-recapture (CMR) methods have frequently been applied for estimating animal population sizes [1,2,3]. Today, molecular techniques allow extraction of DNA from material dropped by animals (hairs, feathers, droppings) to distinguish individuals based on their genotypes [4,5,6]. Repeated samples of genetic material effectively represent capture-recapture samples and can thus be analyzed with statistical models analogous to classical CMR, if the particular problems associated with non-invasive genetic sampling [7,8,9] are considered.

In conservation biology, one often has to work with small and potentially biased samples. Small samples can cause imprecise estimates of key population descriptors such as abundance. An important potential source of bias is the widespread occurrence of detection errors: a) rarely every member of a population is detected, and b) different classes of individuals may differ in their detectability. Problem a) leads to underestimation of the population size, while problem b) may lead to distorted assessments of population structure, e.g., in terms of age, sex or life stage [10].

Recently, the spatial attribution that is characteristic of nearly all detection data has been incorporated directly into capture-recapture models to jointly estimate the number *and* the locations of individuals’ activity centers in spatial capture-recapture (SCR) models [11,12,13,14]. These models show promise for improving inferences from sparse encounter history data because they more fully utilize the encounter data compared to classical non-spatial models which typically produce encounter histories by collapsing over space.

The capercaillie (*Tetrao urogallus* L.) is a rare and elusive species of great conservation concern in much of Europe. Reliable information about population status and trends of this species is very hard to obtain in a way that is not potentially harmful to relict populations of this extremely shy species. In Switzerland, capercaillie abundance has traditionally been estimated from lek-based counts of males, observations of the species away from leks and indirect evidence of presence such as droppings, tracks and feathers [15,16]. Based on the assumption of an even adult sex ratio, the number of counted males was then multiplied by two to obtain an estimate of total abundance. However, there are at least two problems with this approach to estimating abundance. First, lek attendance is age-dependent. Males at least three years of age defend territories close to the lek center, while younger males establish peripheral territories or do not show territorial behavior or sometimes do not even show up at leks at all [17,18]. Second, male capercaillies, even older males, do not always form clearly identifiable leks [19,20,21]. Clearly, where there are no leks, lek-based abundance estimation is not possible.

Alternative methods for estimating capercaillie abundance have been described by Rajala [22] and Lindén et al. [23]. These methods rely on transect surveys, where several observers move simultaneously along parallel transects. However, in many areas, including the one of our study, these methods are impracticable because of the rugged terrain. Jacob et al. [24] estimated population size of Swiss capercaillie populations based on a single field sample using the software CAPWIRE [25]. Here, we report on a study that was conducted with a more rigorous field design, higher sampling effort, and an explicitly model-based statistical analysis that yields estimates of both population size and sex ratio. In addition, the analysis applied here can easily cope with samples for which no sex determination was achieved and, importantly and in contrast to non-spatial, traditional capture-recapture methods, can do so with a single sample.

## Methods and Materials

### 2.1 Study site and sampling design

**Fig 1:**
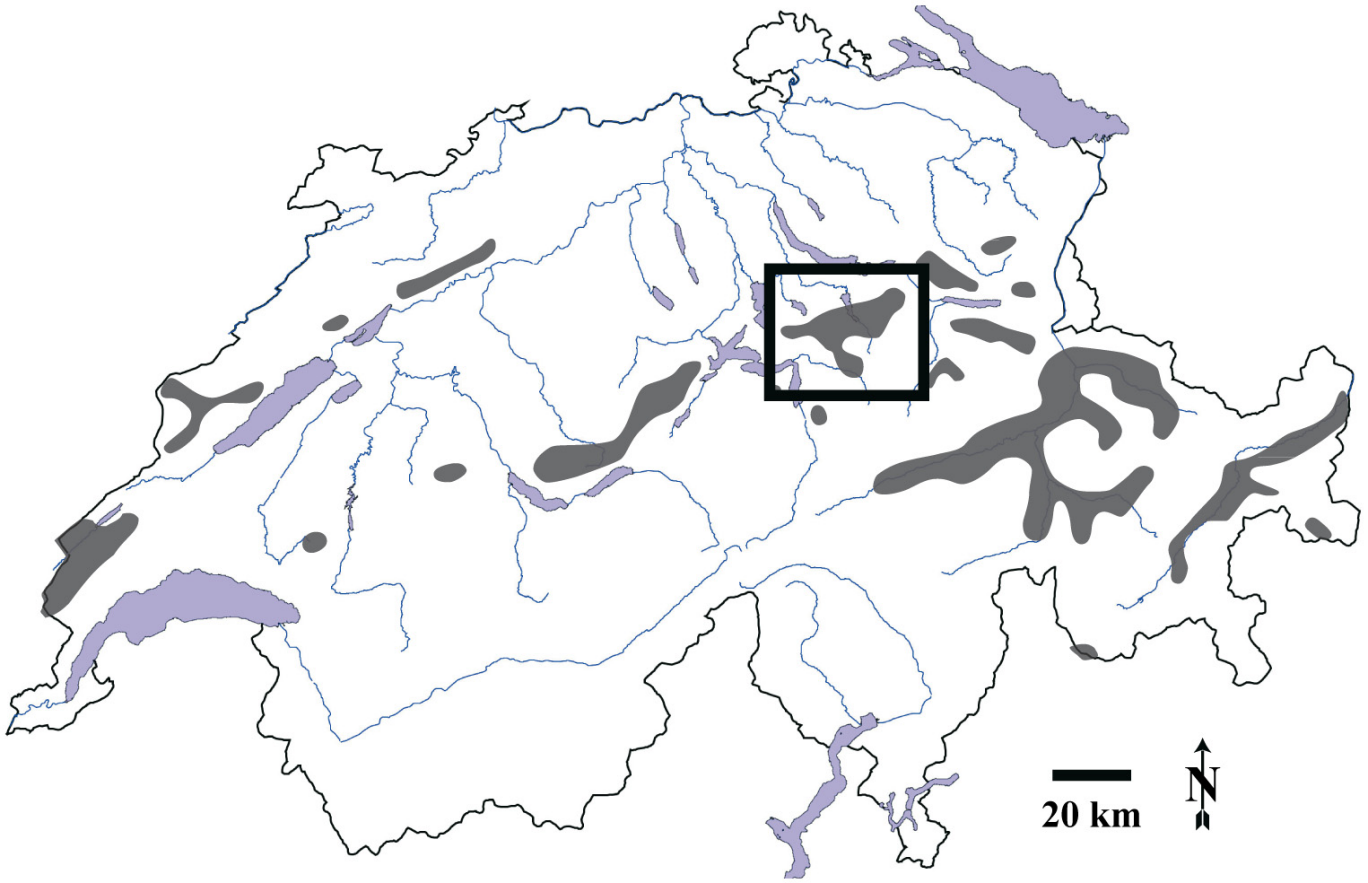
Geographical location of our study area (black rectangle) in Central-Eastern Switzerland. Dark shading shows the current capercaillie distribution range in Switzerland.

Our study area was located in Central-Eastern Switzerland (Fig 1), on the northern slope of the Alps, and consists of several mid-elevation, mostly wooded foothill ranges up to 1600 m above sea level, separated from each other by deep valleys with agricultural land, towns and some small lakes. The forests on the hills are dominated by Norway spruce (*Picea abies* L.) and are interspersed with many small mires and some pastures used for grazing cattle and sheep. Mountain pine (*Pinus mugo* Turra) is an important species in the tree layer in some forests, notably on boggy soils.

**Fig 2:**
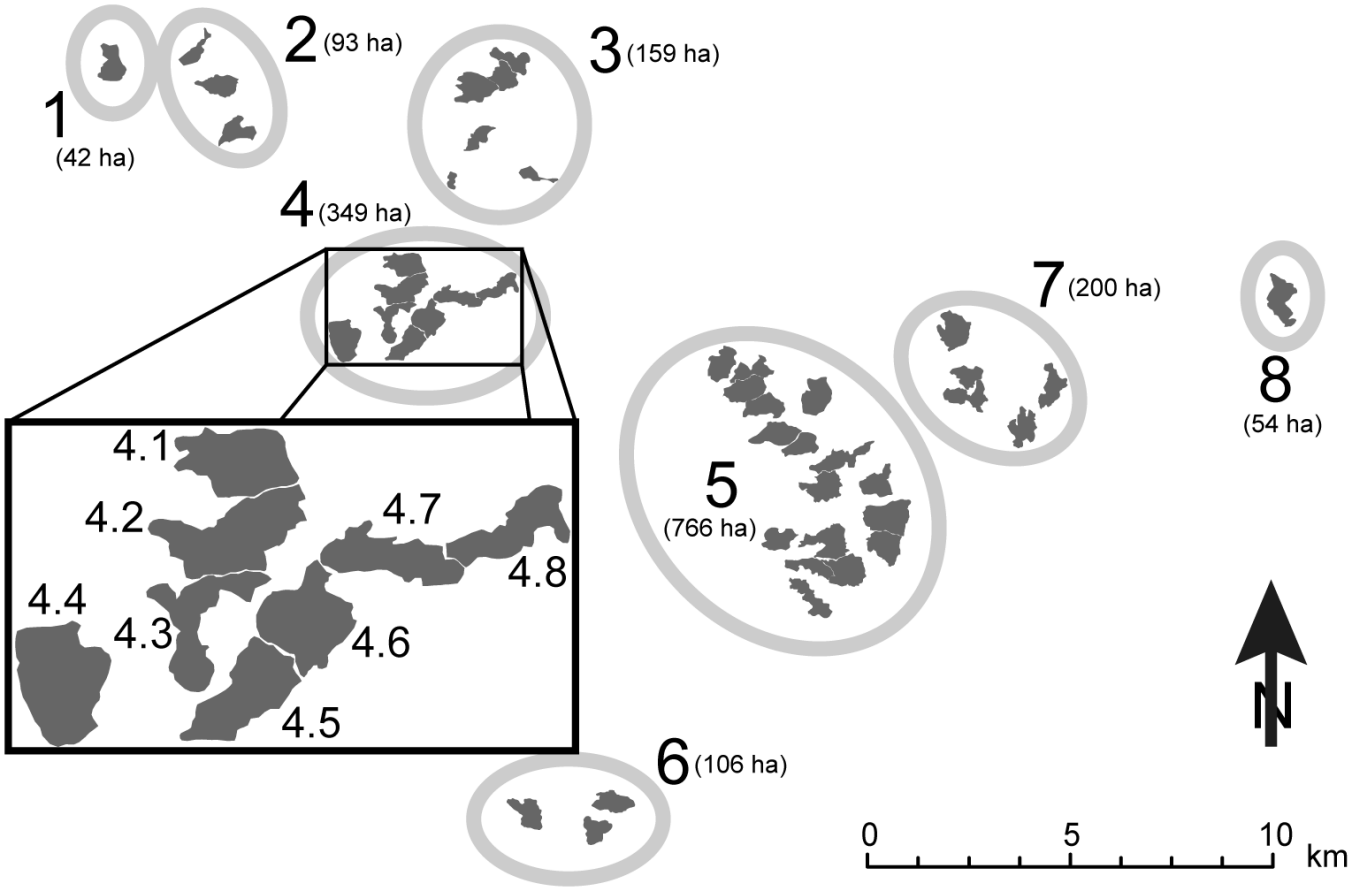
Spatial distribution of the capercaillie habitats within the study area. The eight fragments 1 to 8 are located on eight wooded ranges of hills. Shading within each fragment indicates the capercaillie distribution area. This area is broken down into spatial units (totally 44, of which 39 were surveyed), as exemplified by the inset for area 4. For the statistical analysis, each such unit was further divided into two subunits to improve the spatial resolution of our analysis.

The spatial distribution of capercaillie in the study site is well-known due to regular surveys carried out by local dedicated bird watchers, gamekeepers and foresters. Between 1978 and 2009, more than 1,200 opportunistic observations of capercaillie have been made and registered in databases of the Swiss Ornithological Institute and in data collections of local species experts. In the study area, the capercaillie inhabits eight spatially distinct fragments that are between 2 and 6 km from one another (Fig 2). Within each of these fragments, we defined the capercaillie distribution area considered in this study as follows: around all capercaillie observations made between 1978 and 2009, we drew buffers of 500 m width in a GIS and dissolved their borders to create a single polygon. Single observation points whose 500 m-buffers did not interfere with the buffer of any other point were considered isolated observations of non-resident birds and were discarded. We then clipped the resulting surface with the GIS-layer of the forested surface according to the Swiss topographic map (www.swisstopo.ch). The resulting area was defined as the capercaillie distribution area considered in this study, encompassing 1,769 ha.

We divided this capercaillie distribution area into 44 units (Fig 2), considering the following criteria: for executing the sampling in the field, 22 (including the first author) experienced volunteers were available and each of them was prepared to invest up to 4 days of field work, which resulted in 88 person-days available to be spread over the total area inhabited by the capercaillie. Thus, for covering the whole surface of 1,769 ha twice (= 3,538 ha), an average of approx. 40 ha per day and volunteer needed to be surveyed. Because orientation in the field is much easier when the limits of the sampling units are clearly visible (following forest limits, creeks, crests, roads or any other well visible lines), we used such clearly visible lines to separate each unit from the neighbouring ones. As a result, the surface of our units ranged between 26 and 70 ha, with an average of 40 ha. Every unit was sampled by one volunteer in one day's work (6 to 8 hours). Within each unit, we focused our search for droppings in the units below roosting trees and feeding trees, in hiding sites, along internal forest edges, around root plates and on tree stumps. These habitat elements are the ones commonly used by the birds in winter and in spring at a small spatial scale [24,26]. We trained all field volunteers during a one-day excursion to look for scat at every single of these habitat elements present in their sampling units. For cases where there were too many of them, field volunteers were instructed to look at every second or every third of these preferred habitat elements.

We collected the freshest single piece of dropping wherever droppings or clusters of them were found. Sometimes, presumably in the vicinity of leks, hundreds of droppings were clustered in a rather small area of 2–3 hectares. In such situations, we collected a fresh piece of dropping every 25 m to avoid collecting too many droppings of the same individual from the same place. For each dropping collected, the exact location was recorded with GPS (Garmin eTrex Legend HCx, accuracy approx. 5 m). Droppings were stored in 50 ml plastic tubes and deep frozen at –25°C on the day of collecting.

Two surveys of a unit were done within 11 days. The first sampling was conducted between 6 and 8 April 2009 and the second between 13 and 17 April 2009. With one exception, each unit was surveyed by different observers on the first and second sampling occasion, respectively, to ensure independent surveys. Five spatial units could not be sampled because of time constraints, resulting in a total of 39 units sampled. Detection probability of the dark-colored droppings is much higher on snow-covered than on snow-free surfaces. To standardize detection probability as much as possible, fieldwork was carried out when the entire study site was still completely covered with snow. Access to fragment 6 (Fig 2) is possible only in late spring. Sampling there started on 20 April, and the three units in fragment 6 could be sampled only once, because rapid snow melting towards the end of April precluded repetition of sampling. To increase the spatial resolution of the analysis, the surveyed 39 units were divided up into two parts, each with approx. half of the area, resulting in 78 subunits to which each capercaillie detections were assigned. This division was done in the GIS-system, drawing a line by hand across every unit.

### 2.2 DNA extraction and genotyping

Extraction of DNA and genotyping of samples were done by Ecogenics GmbH in Zürich-Schlieren, Switzerland, following exactly the method described by Jacob et al. [24].

Ten nuclear microsatellite loci developed for the capercaillie [24,27] and two additional nuclear microsatellite loci, BG15 and BG18, developed for the black grouse (*Tetrao tetrix* L.; [28]) have been amplified. Details on forward and reverse primer sequences and repeat motifs are given in Jacob et al. [24]. The twelve microsatellite loci were amplified in four multiplex-PCRs, each containing three primer pairs differing in their fluorescent labeling dyes (FAM, HEX, NED; Applied Biosystems). A fragment of the chromo-helicase-DNA-binding (CHD) gene using the primer pair P2 and P8 of Griffiths et al. [29] was amplified to sex the individual.

Loci that could not be scored after eight PCR replicates were coded as missing values. Samples with a low prospect of producing a multi-locus genotype (no amplification products at any of the three loci) and those assigned to other grouse species were discarded. Capercaillie samples were typed with the nine remaining microsatellite markers, organized in three multiplex-PCRs, and the sex-specific locus following the same genotyping procedure. Based on the analyses by Ecogenics GmbH, only samples with at least 8 loci unambiguously genotyped were retained for further analyses. PI_sib_ (see below) of the 8 least informative loci was below 0.01 (cf. [30].

Two multi-locus genotypes were considered to be identical if they shared all the alleles at all the loci, excluding loci with missing values. To reduce the chance of erroneously considering two genotypes as identical as a consequence of errors in the process of genotyping or recording of the data, those genotypes differing only because of missing values and those differing by a single allele were re-analyzed. An allelic combination representing one or several identical genotypes was considered to be unique if it differed from all the other allelic combinations by at least two alleles (excluding missing values).

Probability of identity (PI) of the genotypes obtained as just described was calculated with Cervus 3.0.3 [31]. Estimation of PI assumes that individuals to be compared are unrelated. Because the samples potentially included relatives, we also calculated PI_sib_, which gives an alternative and more conservative estimate of the probability of identity than PI [30].

Loci were checked for departure of Hardy-Weinberg (HW) expectations using an exact test [32] with 10,000 dememorization steps, 5,000 batches, and 10,000 iterations per batch in GENEPOP on the web (http://genepop.curtin.edu.au; [33,34]). Null allele frequencies (F_Null_) and measures of genetic diversity, i.e. alleles per locus (A), observed heterozygosity (H_o_), and expected heterozygosity (H_e_), were calculated using Cervus 3.0.3. We also computed a rarefied estimate of allelic richness (R) in FSTAT 2.9.3.2 [35], which accounted for sample size differences among loci.

### 2.3 Statistical model

We used spatial capture-recapture (SCR) models to estimate sex- and fragment-specific population sizes and capercaillie sex ratio, accounting for imperfect detection and the spatial attribution of all detections. SCR methods use detection devices (e.g., camera traps, mist nets) to obtain spatial encounter histories or trap-specific encounter frequencies, *y*_*ij*_, for individual *i* and trap *j*. A standard type of model applies to situations in which encounter is a binary event and assumes that *y*_*ij*_ is a Bernoulli outcome with parameter *p*_*ij*_, the encounter probability for individual *i* at trap *j*. The key aspect of SCR models is that encounter probability depends on the location of the trap based on the distance from the trap to the animal’s activity center. Standard SCR models parameterize individual location by a latent variable, say *s*_*i*_, which is interpreted as the home range or activity center of the individual and in SCR models is a random effect with a prior distribution and, in essence, is the realization of a spatial point process [36]. The customary null model is that *s*_*i*_ are independent and uniformly distributed over some prescribed region, *S*, referred to as the state-space, i.e., the range of all possible values *s*_*i*_ can take. The model is therefore analogous to a generalized linear mixed model, and standard classical or Bayesian methods of analysis can be used for parameter estimation and inference. See [37,38,39] for further explanations of spatial capture-recapture models.

For our capercaillie survey, the data collection is not characterized by a set of discrete trap locations as it is customary in, for example, camera trap studies (e.g., [40]). Instead, the data are obtained by people searching each of the 78 sample subunits in a subjectively systematic fashion, as described above. We developed a model in which each of these 78 subunits is regarded as a potential encounter location (effective “traps”), the location of which is characterized by its centroid. Thus, we constructed spatial encounter histories by associating each encounter observation with one of the 78 sample subunits. We assumed the population was closed over the 2 week sample period and pooled the two samples into a single plot-specific encounter frequency for each individual, say *y*_*ij*_ for individual *i* and plot *j*. For numerical reasons, we standardized the metric coordinates by dividing them by 5000.

Individuals may be captured an arbitrary number of times in each sample subunit and so, unlike in traditional capture-recapture studies, the observations in this case are counts with no natural upper-bound. As such, we develop models based on the assumption that *y*_*ij*_ has a Poisson distribution with mean *λ*_*ij*_, which will depend on the location of each sample subunit (represented by the centroid of the patch) as well as the home range center of each individual. Our basic model assumed that

$$\text{log}(\lambda_{ij})=\beta_{0}+\beta_{1}*d^{2}(s_{i},x_{j}),$$

*d* ^*2*^ is the squared distance between *x*_*j*_, the centroid of plot *j*, and *s*_*i*_, the "activity center" of individual *i*, *β*_0_ and *β*_1_ are the intercept and the slope (on squared distance) of the log-linear regression of the expected capture frequencies. The use of the squared distance can be motivated by the assumption of a bivariate normal model for individual movements; see below. Here we assumed also that the state-space (the possible values) of *s*_*i*_ is discrete and associated with one of the 78 sample subunits (i.e., the centroids) in proportion to the surface of the sample unit. Because sample subunits have variable surfaces, we assumed that the prior density of activity centers was in proportion to the surface of each subunit. In the model, this was parameterized by assuming that *s*_*i*_ is a categorical random variable:

$$s_{i}\sim \text{Categorical}(\pi_{1}\ldots \pi_{78})$$

where:

$$\pi_{j}=\text{exp}(A_{j}/\sum_{j}A_{j})$$

is the probability of *s*_*i*_ occurring in subunit *j*. Thus, the prior for the distribution of the locations of the individual home-range centers was proportional to the size of each area, *A*_*j*_.

Our basic model embodies the assumption that the encounter frequency of individual *i* in survey subunit *j* is proportional to the distance between the centroid of the subunit and the activity center. In particular, the logarithm of the expected frequency for the subunit, in which individual *i* has its home range center, is precisely *β*_0_. In our analysis we allowed the parameters *β*_0_ and *β*_1_ to be sex-specific. Because sex is unobserved for individuals that were not captured, we regarded sex as a latent variable with a population proportion to be estimated. Similarly, four individuals could not be sexed. Bayesian analysis excels at doing calculations with latent variables. Hence, it was very easy to estimate unknown sex as an unobserved categorical individual covariate, or a latent variable. Home-range centers, *s*_*i*_, are similar latent variables or random effects, and tallying up their number per fragment is trivial in a Bayesian analysis and enables one to estimate population size per fragment.

For comparison with the SCR model we also analyzed the individual, binary detection/nondetection data for the two occasions under a traditional, non-spatial capture-recapture model. Specifically, we used model M0 which assumes constant detection probability among all individuals and occasions [41]. For both the spatial and the non-spatial capture-recapture model we chose a Bayesian analysis which was carried out in WinBUGS [42,43] using the R package R2WinBUGS [44]. We used a formulation of both models based on data augmentation [45] which facilitates implementation of the model in WinBUGS. See [46,47,48 (chapter 6)] for additional details and S1 Appendix for a description of the model in the BUGS language.

### 2.4 Simulation study to investigate effects of reduced sample size

To study the quality of the estimates of population size and sex ratio under a scenario with reduced sample size, we conducted a ‘data dilution’ simulation experiment. We simulated three cases where effort was reduced such that resulting sample sizes (in terms of the number of individuals detected) were 75%, 50% and 25%, compared to our actual sample size comprising 466 detections for 127 birds. For each scenario, we randomly sampled a proportion of 25, 50 or 75% of the data, fitted our model to the reduced data set and saved the estimates. We repeated this 100 times for each scenario and present the estimates for total population size and for the sex ratio within the studied areas.

### 2.5 Ethics statement

In Switzerland, federal law guarantees free and open access to any land covered by forests or pastures, regardless of whether it is privately or publicly owned. The only exceptions are National Parks and Wildlife refuges, where access is only possible with specific permission. The land accessed in our study was completely outside all National Parks or Wildlife refuges, hence no specific permissions of any kind were required to access the land, nor was it required to know the landowners. Just looking for scat and not for the birds themselves, we a) reduced disturbance of the birds to the possible minimum and b) did not need any specific permission to work with living animals.

Geographical coordinates of capercaillie distribution hotspots, particularly leks, are highly sensitive information, so we did not publish them.

## Results

### 3.1 Genotyping success

In total, 586 droppings were analyzed, of which 69 could not be genotyped, either because DNA could not be extracted or because of poor amplification of several loci in the PCR; of the remaining droppings, 51 were determined to belong to black grouse (*Tetrao tetrix*). This left us with 466 capercaillie droppings to be used in subsequent analyses.

### 3.2 Probability of identity and genetic diversity

The cumulative discriminatory power of the 12 loci to identify individual genotypes was very high (PI = 5.0582e-10 and PI_Sib_ = 1.3403e-04). Two loci deviated from HW expectations after Bonferroni correction (Table 1). The power of each of these two loci to contribute to a unique genotype was relatively low (Table 1). PI and PI_sib_ calculated without these two loci remained high (4.5039e-09 and 3.9130e-04). Finally, the cumulative discriminatory power of the 8 least informative loci to identify individual genotypes was 1.5308E-05 for PI and 6.6087E-03 for PI_sib_, respectively, and thus below the 0.01 usually recommended for population size estimates [30].

**Table 1:** Probability of identity (PI) and measures of genetic diversity at 12 microsatellite loci in our capercaillie population sorted by polymorphic information content (PIC).

Locus	N	A	R	H_o_	H_e_	PIC	PI	PI_sib_	HW	F_Null_
sTuD7-FAM	119	2	2	0.639	0.489	0.369	0.382	0.602	0.001*	-0.135
sTuD3-NED	119	5	5	0.496	0.517	0.444	0.306	0.569	0.076	0.017
sTuT1-VIC	118	4	3.992	0.364	0.511	0.456	0.294	0.569	0.000*	0.154
sTuT3-FAM	120	5	5	0.492	0.527	0.469	0.282	0.558	0.555	0.036
sTuD6-NED	119	12	11.966	0.571	0.528	0.509	0.241	0.548	0.728	-0.061
BG15-FAM	117	5	5	0.607	0.597	0.527	0.232	0.511	0.069	-0.007
sTuT2-VIC	120	5	4.999	0.533	0.586	0.538	0.219	0.513	0.008	0.043
sTuD1-NED	120	6	5.975	0.708	0.722	0.669	0.129	0.423	0.099	0.011
sTuD4-NED	118	8	7.991	0.678	0.741	0.697	0.11	0.409	0.196	0.044
sTuT4-PET	120	5	5	0.683	0.769	0.727	0.093	0.391	0.136	0.055
BG18-FAM	120	7	6.95	0.817	0.781	0.743	0.085	0.382	0.068	-0.025
sTuD5-PET	120	10	9.974	0.833	0.859	0.838	0.038	0.332	0.539	0.012
Across loci		6.167 (0.787)	6.154 (0.783)	0.618 (0.04)	0.636 (0.038)	0.582 (0.042)	5.0582E-10	1.3403E-04		

Across loci = means (and SE) over loci for A, R, H_o_ and H_e_, and combined probability for PI and PI_sib_ over all loci. HW = p-value of exact test [32], with asterisks indicating significant deviation from HW expectations at a Bonferroni-corrected α-level of 0.0042 (p-value of 0.05 divided by 12). N = number of samples, A = number of alleles, R = allelic richness (based on a minimum sample size of 117 individuals), H_o_ = observed heterozygosity, H_e_ = expected heterozygosity, PIC = polymorphic information content, a measure of informativeness related to expected heterozygosity and calculated from allele frequencies [31], PI = probability of identity, PI_sib_ = probability of identity considering the possible presence of siblings (see [30]), F_Null_ = Frequency of null alleles per locus; negative values indicate excess of observed heterozygote genotypes.

All 12 loci were polymorphic (Table 1). The mean number of observed alleles (A) and mean allelic richness (R) were very similar (6.17 and 6.15) as were mean H_o_ and mean H_e_ (0.618 and 0.636).

### 3.3 Population size and adult sex ratio

**Fig 3:**
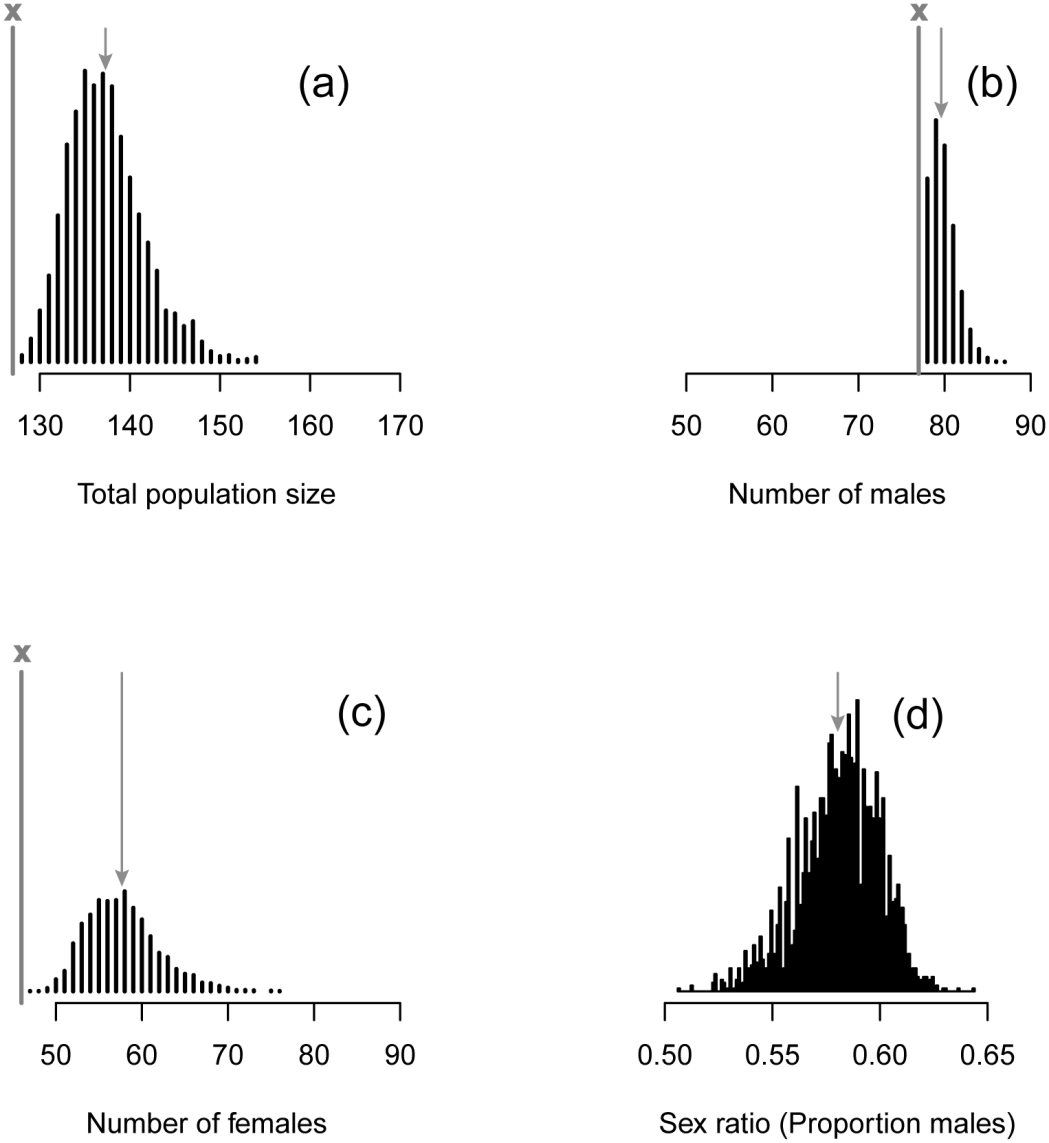
(a) Posterior distribution of the total Capercaillie population size in the study area based on the SCR model. (b) Posterior distribution of the number of males. (c) Posterior distribution of the number of females. (d) Posterior distribution of the sex ratio (proportion males). Grey line with x: observed numbers of individuals, grey arrow: posterior means. The y axis shows the density.

**Fig 4:**
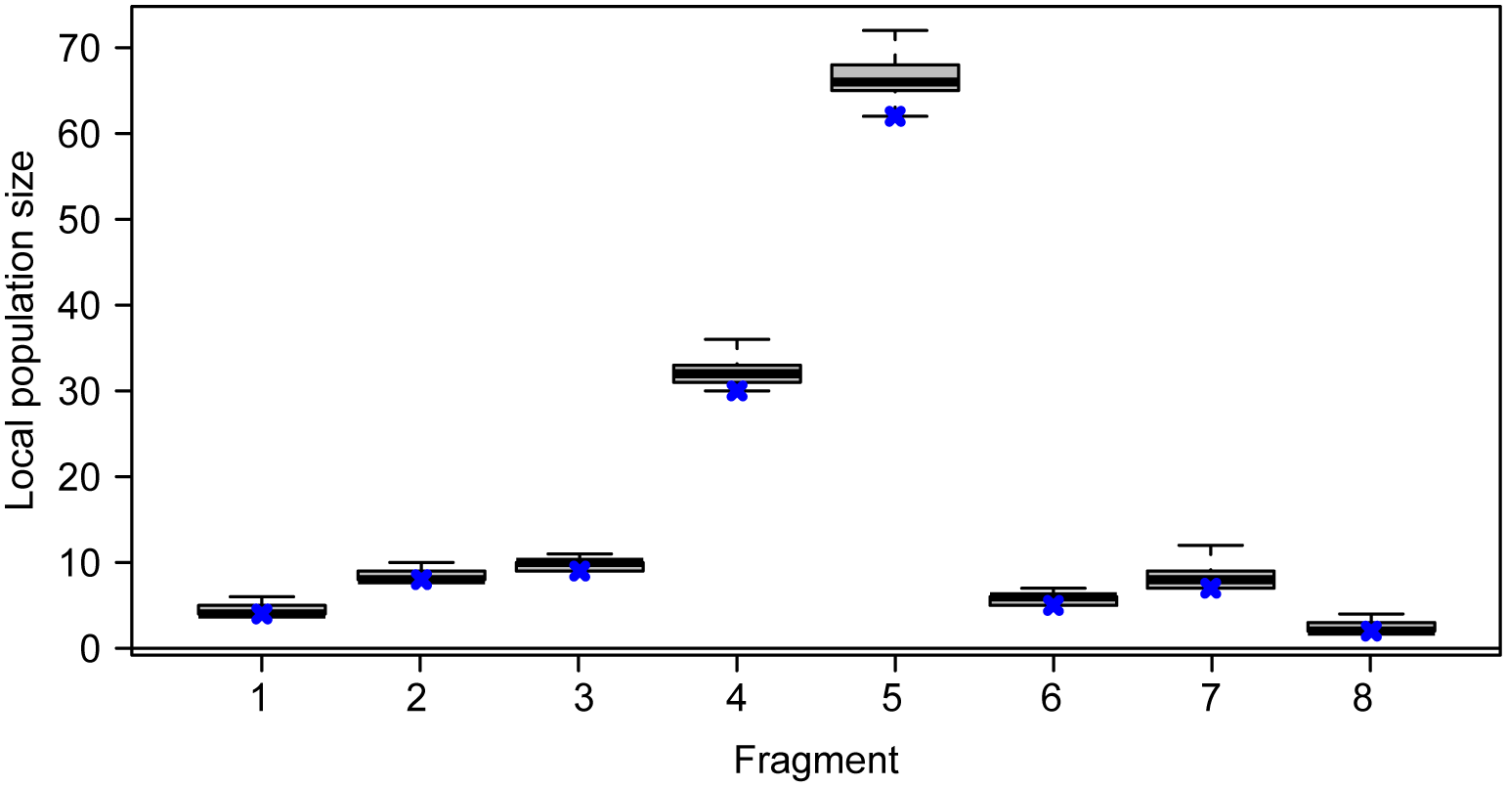
Posterior distribution of the local population size in each fragment based on the SCR model. Boxplots indicate first, second (median, thick line) and third quartiles and the central 95% percentiles. Black crosses denote the observed number of capercaillies in each fragment.

A total of 127 capercaillies were identified: 77 males, 46 females and 4 of unknown sex. Fitting the traditional (non-spatial) CMR model M0 to the raw detection data the estimate of total population size (posterior mean) was 137.0 (posterior sd 4.5, 95% CRI 130–147). On fitting the spatial capture-recapture model (see posterior summaries in Table 2), we estimate a total population size (posterior mean) in the study area of 137.3 capercaillies (posterior sd 4.2, 95% CRI 130–147, Fig 3a). Population size was largest in fragments 5 and 4 respectively (Fig 4).

**Table 2:** Posterior summaries for key quantities in the population analysis of Schwyz capercaillies.

	mean	sd	2.5%	25%	50%	75%	97.5%	Rhat	n.eff
sigma[1]	0.077	0.006	0.066	0.073	0.077	0.080	0.089	1.001	3000
sigma[2]	0.108	0.004	0.100	0.105	0.108	0.110	0.116	1.001	3000
beta0[1]	0.511	0.073	0.383	0.460	0.507	0.560	0.662	1.001	3000
beta0[2]	0.795	0.056	0.688	0.755	0.792	0.832	0.909	1.001	3000
probmale	0.579	0.045	0.491	0.551	0.579	0.610	0.666	1.001	2200
N	137.287	4.196	130.000	134.000	137.000	140.000	147.000	1.001	3000
Nmales	79.616	1.618	77.000	78.000	79.000	81.000	83.000	1.002	1500
Nfemales	57.671	4.139	51.000	55.000	57.000	60.000	67.000	1.001	3000
SR	0.580	0.019	0.540	0.568	0.581	0.594	0.612	1.001	3000

Sigma = parameter that determines the decline of detection frequency of an individual with increasing distance of its activity center from the half-unit center (“the trap”) beta0 = baseline frequency of detections. Indices 1 and 2 denote females and males, respectively. Probmale = sex ratio in a wider statistical population of capercaillies, from which the SZ population can be regarded as a random sample. N = total population size of capercaillies, i.e., the number of capercaillies that were exposed to sampling within the surveyed units. Nmales and Nfemales = estimated number of males and females among N. SR = sex ratio in N.

The number of males was estimated at 79.6 (posterior sd 1.6, 95% CRI 77–83; Fig 3b) and that of females at 57.7 (posterior sd 4.1, 95% CRI 51–67; Fig 3c). Based on our model, 97% of all males were detected at least once (77/79.6), but only 80% of females (46/57.7) were ever detected. Accordingly, there was much more uncertainty in the population size estimate of females. The observed adult sex ratio in our sample was male-skewed (62.6% males observed). The estimated population sex ratio (posterior mean and sd) was 0.58 (0.02, 95% CRI 0.54–0.61), slightly lower than observed due to the apparent sex difference in encounter rate (see below). That the posterior distribution did not overlap 0.5 (Fig 3d) represents strong evidence for a male-biased sex ratio in this population.

The estimate (posterior mean with sd) of the baseline encounter rate *β*_0_ was 0.51 (0.07) for females compared with 0.80 (0.06) for males. Hence, females were much more elusive, because they were estimated to produce only 0.51 detections as a baseline compared to 0.80 for males. In addition, the droppings of individual females were much less widely dispersed than those of individual males, as indicated by the sex-specific differences in the estimates of the SCR model scale parameter *σ* (Table 2). Hence, based on the locations of droppings, females ranged much less widely and produced fewer detections than males, and both contributed to the lower detection probability of females.

### 3.4 Simulation study to investigate effects of reduced sample size

**Fig 5:**
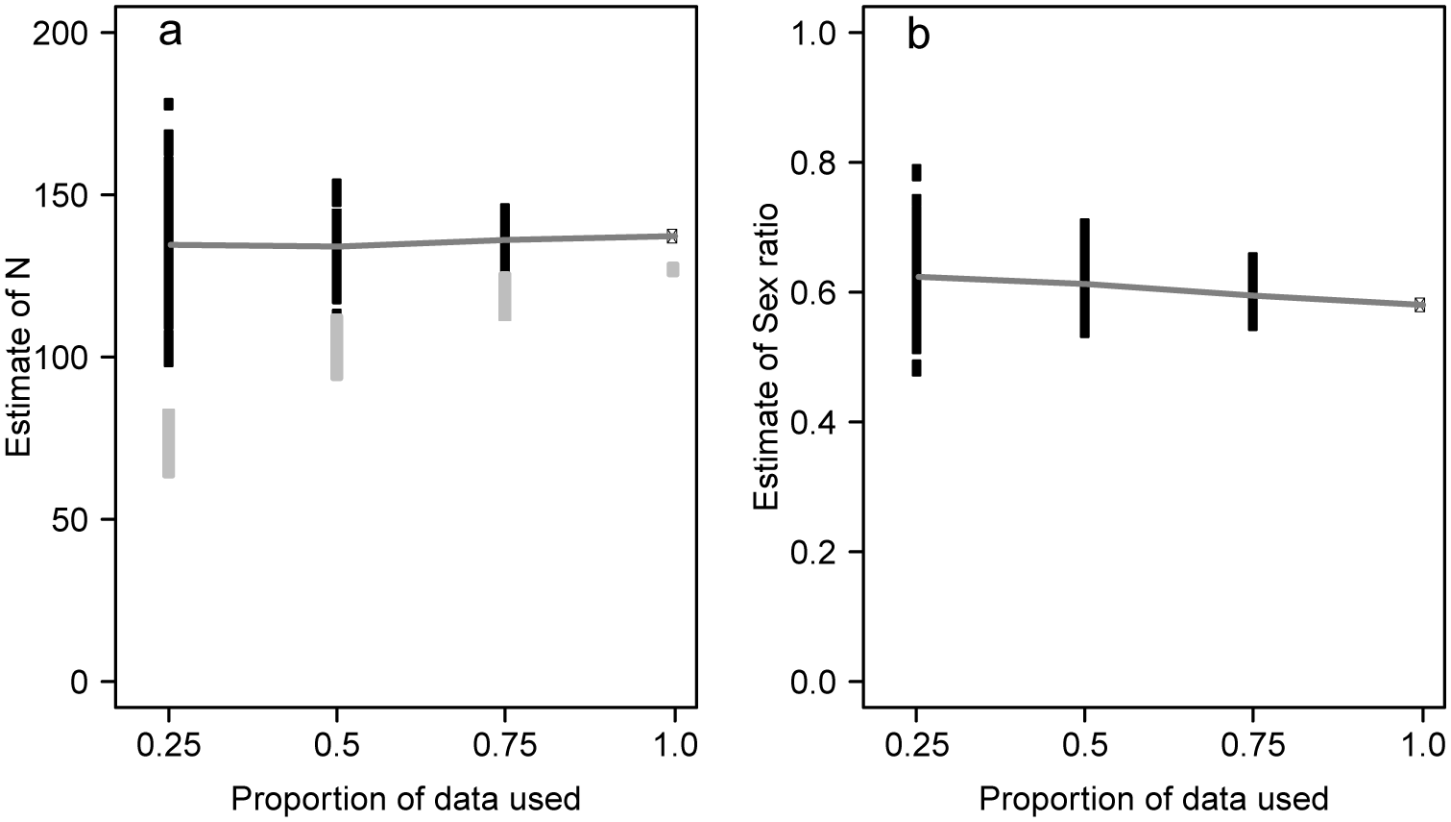
Simulation-based assessment of the effects of reducing the sample size (total number of analysed droppings) from the actual 466 to 75%, 50% and 25% on estimates of (a) total population size and (b) sex ratio. For each scenario, 100 replicate subsamples from the 466 detections were drawn and the resulting data set analysed using our model. The grey horizontal line links the mean for each scenario and the estimate in the actual data set (proportion of data used: 1.0). The grey symbols in panel a indicate the observed number of individuals.

Our simulation suggested that the estimator of total population size was not biased even when only 25% of the actual data were modeled (Fig 5a). However, the precision of the estimates declined drastically when only 25–50% of detections were modeled. In contrast, the non-horizontal grey line in Fig 5b suggested that the sex ratio estimator may be slightly biased for sample sizes of only 25–50% of the actual data set. Altogether, our simulation results suggest an adequate estimator quality would have been obtained with only 75% of the actual data set.

## Discussion

### 4.1 Population size and adult sex ratio

With the kind of spatial capture-recapture model developed for this study, accurate population size estimates for such elusive species as the capercaillie can be obtained with data from just one sampling occasion, provided that at least some individuals are detected at multiple locations. The approach helps to greatly reducing the costs and simplifying the organization of field work compared to more classical capture-mark-recapture statistics which require at least two sample occasions. In addition, sampling only once makes any trap-response impossible, i.e., the tendency for an individual previously detected to be more or less likely to be detected again [41]. Such trap response can easily arise by either the behavior of the animals or by observers remembering where they detected animals previously and must be modeled to prevent bias.

Population size of capercaillie in the study area was last estimated in 2001, when a nationwide capercaillie count based on censuses of lekking males was carried out [16]. The number of lekking males was estimated between 42 to 50 and, assuming a sex ratio of 1:1, a total population size of 84 to 100. Although we do not have data for population trends between 2001 and 2009, we are convinced that, similar to other studies like [24], our results show that expert knowledge based on lekking males’ data underestimates population size.

The population size estimates obtained with the SCR model are almost exactly the same as those under a model M0 fitted to the raw detection data (posterior means 137.3 vs. 137.0, posterior sd's 4.2 vs. 4.5, 95% CRI's 130–147 vs. 130–147). However, while the point estimates of population size are similar, the estimate obtained under the SCR model always applies to an explicit area, and thus the estimate so obtained also yields directly a density estimate. Conversely, ordinary capture-recapture models do not normally allow one to estimate density in a formal way, because the effective sample area is unknown [39]. In the present case, however, we feel confident that the area over which the population exists is well-defined (by the sampled surfaces) and so this is a rare situation in which the population size under the classical closed population models has essentially the same meaning as under the spatial capture-recapture models.

We have not found any obvious explanation for the much lower detection probability of females compared to males. Since we sampled virtually the whole habitat regularly used by capercaillie in our study areas, it is unlikely that many females avoided our sampling area. We rather think that behavioral differences led to the difference in detection probability between the sexes. Females are probably moving less conspicuously and are looking for cover against predators more constantly than males, because they are exposed to more potential predator species due to their smaller body size. Hence, females probably spread their droppings over smaller surfaces, which would let them go undetected with the sampling intensity applied in this study. This hypothesis could be tested by sampling at a substantially increased intensity.

Donald [49] found an increase in adult sex ratio skew towards males with IUCN threat status categories. Our findings are in line with this general relationship, as capercaillie is considered endangered in Switzerland [50]. In NW Spain, too, Morán et al. [51] found such a sex ratio skewed towards males in a highly endangered population of the subspecies *T*.*u*. *cantabricus*. However, the mechanisms underlying this relationship are not clear. Regardless of the mechanism, the male-skewed sex ratio found in our study could be critical because theoretical models suggest that in polygynous species, as for example in the capercaillie, extinction risk is lowest when the adult sex ratio is female-skewed [52]. We acknowledge, however, that data from additional years are needed from our population to confirm the male-biased sex ratio, because sex ratios may show considerable temporal variation (e.g. [53]).

### 4.2 Simulation study to investigate effects of reduced sample size

The main disadvantages of the method developed in our study are the still considerable costs of the genetic analyses that might prevent the approach's regular application. However, our dilution simulation suggested that field and lab efforts might be reduced by up to 25%, yielding a sample only 75% of the size of our actual data set (n = 466 droppings), and estimates of total population size and of sex ratio that still appeared sufficiently accurate to us would still have resulted. This result could not have been anticipated without collecting and analyzing a high number of droppings, but may suggest considerable savings of time and money for repeated inventories of this or similar populations

### 4.3 The statistical model and possible extensions

One advantage of our model as implemented in the Bayesian framework of inference is that latent structure can easily be handled. Thus, sex was not known for all detected individuals, and yet, in the population model these undetermined animals could be modeled jointly with those of known sex and the estimates of all quantities took full account of this added uncertainty in the modeled system. The integration of partial data is much more challenging with an estimation framework based on likelihood as separate likelihood calculations would have to be done for each observation depending on whether sex was observed or not and readily available software implementations to facilitate this had not existed until recently [47]. Similarly, population size in each fragment could easily be estimated by tallying up the number of estimated activity centers, *s*_*i*_, that fall within each fragment; and this would again be much more difficult with non-Bayesian analyses.

There have been a number of recent papers that dealt with capture-recapture data where observations of individuals were made in continuous space as in our study here. Royle and Young [13] considered one case that involved exhaustive searching of spatial polygons with continuous locations recorded within the quadrat, and they specified a continuous space model for the observations. Similarly, Royle et al. [14] used continuous space observations of individuals collected by transect sampling to estimate parameters of an explicit movement model. As in these two studies, our capercaillie data set included continuous space measurements of locations but we did not have precise knowledge of how each sample unit was sampled (the search path) and so we discarded the finer scale information and just associated each observation with the patch centroid. Similar ideas were applied in the recent papers by Thompson et al. [54] and Russell et al. [55] using area searches by dogs to locate individuals or scats used to produce individual level encounter histories. While these two papers addressed a sampling structure similar to Royle et al. ([14]; sample routes digitized with GPS and continuous space observations), the sample routes in both cases were unstructured as dog crews responded to the environment (in Royle et al. [14], the sample routes were fixed a priori). In general, prior expectations regarding important habitat variables could be modeled in the probabilities of the categorical random variable. For example, if a habitat quality measurement was made, e.g., *x*_*j*_ = suitability of sample subunit *j*, then we could model the multinomial logit of *π*_*j*_ as:

$$\text{mlogit}(\pi_{j})=A_{j}+\alpha x_{j}$$

However, in our analysis we do not have additional habitat information.

### 4.4 Recommendations for monitoring

Our statistical approach, combined with the spatially explicit field sampling presented in this study, allows regular accurate estimates of capercaillie population size as an important base of a species monitoring scheme. We feel confident that the approach presented here allows accurate population size estimation for elusive species of conservation concern. However, in situations with lower population densities with an elevated probability of finding many individuals just once, the applicability of this approach still needs to be addressed.

## Supporting Information

S1 Table Locations of all 466 capercaillie samples with sex and consensus genotype. (XLSX)

S2 Table Subunit's centroids locations. (XLSX)

S1 File ReadMe. (PDF)

S1 Appendix Description of our model in the BUGS language [42]. (DOCX)
