# Supplementary material for: Estimating Population Size for Capercaillie (Tetrao urogallus L.) with Spatial Capture-Recapture Models Based on Genotypes from One Field Sample
Source: PLoS One. 2015 Jun 18;10(6):e0129020. doi: 10.1371/journal.pone.0129020 (PMC4472805; doi:10.1371/journal.pone.0129020)
Supplement: S1 File — (PDF) [file pone.0129020.s003.pdf]

Estimating population size for capercaillie (*Tetrao urogallus* L.) with spatial capture-recapture models based on genotypes from one field sample

Pierre Mollet, Marc Kéry, Beth Gardner, Gilberto Pasinelli, J. Andrew Royle
DOI: 10.1371/journal.pone.0129020

Supporting Information

S1 Table: Locations of all 466 capercaillie samples with sex and consensus genotype
This table contains the locations where our capercaillie samples were found. Identified genotypes in the table (tu001, tu002, …) correspond to the individuals i in our model.

S2 Table: Subunit's centroids locations
This table contains the locations of the "traps" (j) in our statistical model.

Coordinates X and Y of both tables are in Swiss Grid (CH1903) coordinates.
