# Supplementary material for: Estimating Population Size for Capercaillie (Tetrao urogallus L.) with Spatial Capture-Recapture Models Based on Genotypes from One Field Sample
Source: PLoS One. 2015 Jun 18;10(6):e0129020. doi: 10.1371/journal.pone.0129020 (PMC4472805; doi:10.1371/journal.pone.0129020)
Supplement: S1 Appendix — (DOCX) [file pone.0129020.s004.docx]

**Appendix**

Description of our model in the BUGS language [42]

model {

**# Priors**

probmale~dunif(0,1)

for(i in 1:2){

beta0[i]~dunif(0,5)

logbeta0[i]<-log(beta0[i])

sigma[i]~dunif(0,5)

}

for(j in 1:ntraps){

log(theta[j]) <- log(fragarea[frag[j]])

probs[j]<-theta[j]/sum(theta[])

}

upper<-ngrid+0.49

psi ~ dunif(0,1)

**# Likelihood**

for(i in 1:M){

w[i]~dbern(psi) # Indicator of ‘existence’

sexmale[i] ~ dbern(probmale) # Potential males

males[i] <- w[i] * sexmale[i] # Realized males

sexfemale[i] <- 1-sexmale[i] # Potential females

females[i] <- w[i] * sexfemale[i] # Realized females

s[i]~dcat(probs[])

x0g[i]<-Sgrid[s[i],1]

y0g[i]<-Sgrid[s[i],2]

for(j in 1:ntraps){

Y[i,j]~ dpois(mu[i,j])

mu[i,j]<-w[i]*lambda[i,j]

lambda[i,j] <- exp(loglambda.lim[i,j])

loglambda.lim[i,j] <- min(999, max(-999, loglambda[i,j]))

loglambda[i,j] <- (1-sexmale[i])* (logbeta0[1] - (1/(2*pow(sigma[1],2)))*pow(x0g[i] - grid[j,1],2) - (1/(2*pow(sigma[1],2)))*pow(y0g[i] - grid[j,2],2) ) + sexmale[i]* (logbeta0[2] - (1/(2*pow(sigma[2],2)))*pow(x0g[i] - grid[j,1],2) - (1/(2*pow(sigma[2],2)))*pow(y0g[i] - grid[j,2],2) )

}

}

**# Population size and other derived quantities**

Nmales <- sum(males[1:M]) # Realized number of males

Nfemales <- sum(females[1:M]) # Realized number of females

N<-sum(w[1:M]) # Total population size

SR <- Nmales / N # Male sex ratio

}
